# Elfin UI: A Graphical Interface for Protein Design With Modular Building Blocks

**DOI:** 10.3389/fbioe.2020.568318

**Published:** 2020-10-23

**Authors:** Chun-Ting Yeh, Leon Obendorf, Fabio Parmeggiani

**Affiliations:** ^1^School of Chemistry and School of Biochemistry, University of Bristol, Bristol, United Kingdom; ^2^Institute of Chemistry and Biochemistry, Freie Universität Berlin, Berlin, Germany; ^3^Bristol Biodesign Institute and BrisSynBio, a BBSRC/EPSRC Synthetic Biology Research Centre, University of Bristol, Bristol, United Kingdom

**Keywords:** protein design, blender, GUI, repeat proteins, computational modeling

## Abstract

Molecular models have enabled understanding of biological structures and functions and allowed design of novel macro-molecules. Graphical user interfaces (GUIs) in molecular modeling are generally focused on atomic representations, but, especially for proteins, do not usually address designs of complex and large architectures, from nanometers to microns. Therefore, we have developed Elfin UI as a Blender add-on for the interactive design of large protein architectures with custom shapes. Elfin UI relies on compatible building blocks to design single- and multiple-chain protein structures. The software can be used: (1) as an interactive environment to explore building blocks combinations; and (2) as a computer aided design (CAD) tool to define target shapes that guide automated design. Elfin UI allows users to rapidly build new protein shapes, without the need to focus on amino acid sequence, and aims to make design of proteins and protein-based materials intuitive and accessible to researchers and members of the general public with limited expertise in protein engineering.

## Introduction

Visualization and simulation of macromolecules have enabled our understanding of biological structures and have led to the development of a variety of tools for research, teaching and outreach, working at multiple scales (Johnson and Hertig, 2014).

Visualizing structures made also possible to design them, by taking into account the spatial relationship between different parts of the molecules. Dedicated software packages have emerged over the years for protein design, reviewed by Gainza et al. (2016), and popular viewers such as Chimera (Pettersen et al., 2004), PyMOL (The PyMOL Molecular Graphics System, Version 2.0 Schrödinger, LLC) (DeLano, 2002), and VMD (Humphrey et al., 1996) have now integrated design capabilities.

Protein design tools focus largely on atomic models and sequence design from a given backbone structure. Additionally, several approaches allow to build completely new structures by relying on secondary structure description and fragments assembly, like Rosetta remodel and blueprint builder (Huang et al., 2011; Koga et al., 2012), parametric design, as in Isambard (Wood et al., 2017), idealized secondary structures, e.g., CoCoPOD (Ljubetič et al., 2017) and TopoBuilder (Sesterhenn et al., 2020), or building blocks with super-secondary structures, as in SEWING (Jacobs et al., 2016) and Elfin (Yeh et al., 2018). Protein complexes have been successfully designed for symmetric systems, e.g., point group symmetry (Lai et al., 2012; King et al., 2014; Hsia et al., 2016) and lattices (Lanci et al., 2012; Gonen et al., 2015), but large, precise and asymmetric assemblies are still a challenge. However, such scaffolds could prove particularly interesting in modulating cell surface receptor clustering and signaling via precise ligand organization and placement (Grochmal et al., 2013; Jost et al., 2013; Shaw et al., 2014; Mohan et al., 2019).

To address the challenge of building large structures, both symmetric and non-symmetric, DNA nanotechnology groups have led the way in developing Computer Aided Design (CAD) software, e.g., Tiamat (Williams et al., 2009), cadnano (Douglas et al., 2009), CanDo (Veneziano et al., 2016), vHelix (Benson et al., 2015), taking advantage of base pairing and regularity of DNA double helix structure.

Graphical User interfaces (GUI) have indeed a key role in making software accessible to a broad group of users, who are not necessarily expert, by enabling work on design principles, rather than biochemical details. While CAD tools for DNA nanostructures allow users to work purely on intuitive geometric concepts, e.g., shapes to achieve, protein design tools often require a more in-depth programming and biochemical knowledge. GUIs have been developed for the Rosetta modeling suite to improve usability (Adolf-Bryfogle and Dunbrack, 2013; Schenkelberg and Bystroff, 2015) and the protein folding game Foldit (Cooper et al., 2010) has successfully attracted a broad base of users from the general public. Its standalone interface (Kleffner et al., 2017) has become an instrument to interactively design new proteins, although designs are effectively limited to a few hundred amino acids, if systems are not symmetric.

Size is one of the major limitations in interactive protein design using atomic models, as the number of atoms quickly becomes the computational bottleneck. However, it is possible to take a more coarse-grained approach to design large and complex protein architectures, akin to DNA nanostructure designs.

**FIGURE 1:**
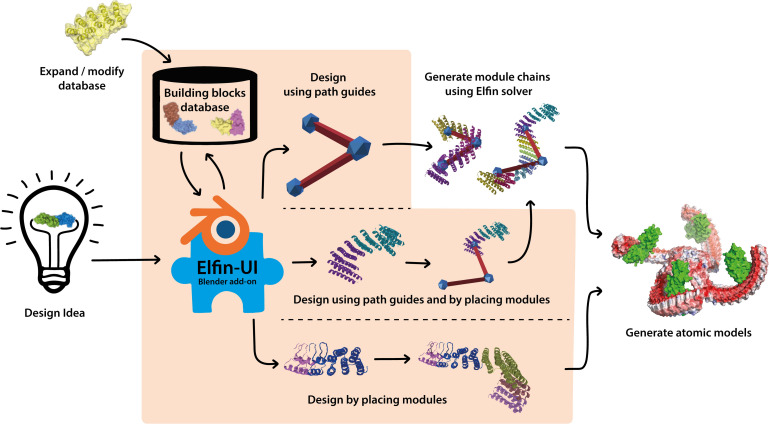
Elfin UI is a Blender (blender.org) add-on that enables interactive coarse-grained design of proteins using combinations of pre-existing and validated building blocks. The shaded orange area indicates the functionalities of Elfin UI within the design process. Designs can be built by defining the desired shape and searching for matching building blocks combinations, by manually placing the building blocks, or by a combination of the two methods. Coarse grained representations are then converted to atomic model outputs in mmCIF format.

In this work we have developed a user interface to allow design of protein structures using modular structural building blocks. Elfin user interface (Elfin UI) was developed as a graphical interface and an interactive editor to the Elfin software package (Yeh et al., 2018) for design of custom protein architectures (Figure 1). Elfin uses structural compatible building blocks (referred to as modules) derived from experimentally validated structures of repeat proteins to build large and complex architectures. The goals were to provide (1) a CAD-like environment for design of user-defined shapes, to which Elfin could find solutions in terms of protein sequence and structures, and (2) a sandbox framework to interactively explore potential protein architectures. We envision Elfin UI to be used in the design of protein origami, custom shaped nanoparticles and scaffolds for organization of enzymes and signaling molecules.

We have implemented Elfin UI as a Blender add-on. Blender is a popular free, open source and cross-platform 3D modeling application, which has been successfully extended with add-ons to integrate molecular viewers, like BlendMol (Durrant, 2019), BioBlender (Andrei et al., 2012), ePMV (Johnson et al., 2011), Pyrite (Rajendiran and Durrant, 2018).

By using modular compatible building blocks and a coarse-grained representation, we aim to provide a tool accessible to scientists, both expert and novice in protein design, and a new way to engage the public with the concepts of modular design and manufacturing using biological macromolecules.

## Methods

The Elfin software package is built around the Elfin solver, a genetic algorithm for the assembly of modular structures matching a user defined shape (Yeh et al., 2018), and contains an updated database with information about modular building blocks, a graphical user interface (Elfin UI) built as Blender add-on, and ancillary utility scripts (e.g., for installation, database preparation, file conversion). Code, documentation, installation scripts and tutorials are available on https://github.com/Parmeggiani-Lab/elfin.

Elfin UI’s approach to protein design is similar to the idea of Model-Based UI Design (Calvary et al., 2003). In this framework, Elfin UI uses a database of individual proteins and termini compatibility matrix as the domain model. The task of protein design is undertaken by arranging and joining two or more protein modules to form the shape desired by the user. Each protein module is abstractly represented by attributes such as its center-of-mass, collision radius, and module pairwise transformation matrices. A design assembled by the user is converted into an atomic model by projecting atomic coordinates of each protein module onto their respective position and adding capping modules to each “free” termini to protect the otherwise exposed hydrophobic core and improve solubility (Supplementary Figure S1). Finally, if the designed protein’s atomic structure passes third party verification (e.g., Rosetta, see Supplementary Materials), it is considered suitable to be produced and characterized experimentally.

### Database

Elfin builds protein architectures using combinations of structural building blocks. Building blocks are stored as collection of atomic coordinates in The Protein Data Bank (PDB) format and used to precompute: (1) a JSON database, which includes, for each module, the center of mass and radius, a list of compatible modules and relative orientation of the pairs, expressed as rigid body transforms; (2) a Blender database that stores meshes of each module with cartoon representations of secondary structure elements.

Modules are classified as: core, when they are extracted from designed repeat proteins (Parmeggiani and Huang, 2017) and contain repeated super-secondary structure (e.g., helix-loop-helix-loop); junction, if they contain two contiguous and merged super-secondary structures typical of core modules (so that the module acts as a junction between core modules); or hub, if they are formed by multiple interacting chains. Core and junction modules are single chains that can be extended by adding a further module to the chain either at the N- or C- terminus. Some hubs’ chains can be extended only at one terminus, if the other is involved in binding another chain.

Core modules have a specific name, like D4, proA, darp. Junctions include the name of the core modules that they bridge with a j (for junction) followed by a number, since there can be multiple junctions between two core modules: e.g., D14_j1_D79, D14_j2_D79. The name indicates that they are compatible at the N-term with modules that possess a C-term interface of the same kind (anything ending in D14 in this case). Same for the C-term. Core modules are compatible, by definition, with themselves and with junctions with compatible ends. Hub names indicate the type of core module that they contain and eventually information about the number of subunits and type of symmetry, e.g., D4_C4 is a cyclic homo-tetramer of D4-derived units.

Modules form a continuous hydrophobic core that runs through each chain. As for repeat proteins (Parmeggiani and Huang, 2017), the core needs to be sealed off at the termini by modified repeating units, called capping repeats or caps, with the same structural unit of the last module: e.g., a D14 and a D49_j1_D14 module, placed at the C-term, require capping by Ccap_D14. Caps are added only at the final stage when a JSON file from Elfin UI or Elfin solver is converted into an atomic model in mmCIF format by the stitch.py script. mmCIF is the standard format for the Worldwide Protein Data Bank (wwPDB) and removes limitations on the number of atoms and chains present in the previous PDB format. Modules in the database are still stored as PDB files, as the number of atoms is limited and within the capacity of the format.

Current modules are derived from published and experimentally verified structures. Core modules are extracted from designed helical repeats (DHRs) (Brunette et al., 2015), designed ankyrin repeats proteins (darpins) (Kramer et al., 2010) and protein A (Youn et al., 2017). Junctions were designed using either an helix fusion method (Wu et al., 2017; Youn et al., 2017) or *de novo* connecting helices (Brunette et al., 2020). Hubs were derived from oligomeric repeat proteins (Fallas et al., 2017). Supplementary Table S1 contains a detailed list of modules and sources. Custom databases can be created using the scripts provided with the Elfin source code. The workflow is described in the Supplementary Figure S2.

### Blender Add-On Implementation

Elfin UI was developed in python 2.7 as an add-on to blender 2.79. Currently it is not yet compatible with Blender 2.8. As Blender add-on, Elfin UI creates a context menu and adds sections in the side panel, but primarily interacts with objects in the scene by defining “operators” that apply some routine on selected objects. These operators can be invoked using shortcuts, by clicking context menu buttons, or looked up and called from the search menu. Elfin UI plugin defines many such operators to facilitate two main design processes: (1) path guide building, and (2) manual module placement (see results for description). Whenever objects (either protein modules or path guide components) are created through Elfin UI’s operators, the object is spawned with a property group dedicated to storing Elfin’s information. It stores the object type (module or path guide), link occupancy (who are the neighbors), and helper attributes such as a flag to indicate whether the object needs to be cleaned up by Elfin’s object lifetime watcher. Other than object-specific information, data such as module compatibility and 3D models are loaded only once and stored in a singleton object until either Blender is closed, the add-on is reloaded, or when the user explicitly calls the reload operator.

Module compatibility is explicitly embedded in the prototype naming convention for module operators. Place Module and Extrude Module operators prompt the user with a filtered list of actionable module names (*filtered prototypes*). There could be many modules in a scene, but modules with the same name (e.g., D4.001, D4.002) are of the same prototype (D4). For extrusion, prototypes are filtered by compatibility and also terminus occupancy (i.e., is the N and/or C terminus already occupied?).

For Place Module, the name of each module is bounded by two period marks. These marks make it easy to search the exact module the user is looking for: e.g., a search for.D4 will return all modules with a name starting in D4.

For Extrude Module, names are in the form:

: < chain1 > (< term1 >) - > (< term2 >) < chain2 > : < name2 >.

The chain1 and term1 are chain ID and terminus type of the module being extruded from. The term2, chain2, and name2 are corresponding attributes of the new module to be extruded into. For instance: when D49 is selected and extrusion on the N terminus is chosen, one of the items in the list could be:A(N) - > (C)A:D49_aC2_24. This means the terminus N of chain A of D49 can be extruded and connected to a yet-to-be-added D49_aC2_24 hub. In the latter, terminus C of chain A would be used for this connection. The first letter, if there is one, denotes the C-Terminus chain ID of the extrusion. This is needed because hub modules have more than one chain to extrude to and from. The last letter is therefore the N-Terminus chain ID in the to-be-extruded module.

Groups of modules or path guide primitives are organized in networks that keep track of which modules or path guides are “connected.” Networks are displayed in Blender outliner view. While individual path guide “joints” can be freely rotated and translated, Elfin UI locks individual modules. However, whole networks can be rotated and translated because they preserve the interface relationship of each connected group of modules. Creation and splitting of networks are automatic, and ease processing when exporting. Joining of two networks is also possible, subject to termini compatibility.

When designing using Elfin UI, live collision detection between protein modules can be turned on or off from the left side pane (default shortcut is T). When it is turned on, newly placed protein objects that result in collision will raise a clear warning on screen.

Since Elfin UI supports “partial design”—a design specification consisting of a network of path guide components overlapping manually placed modules, sanity checks such as overlap intention and link availability are conducted behind the scenes.

## Results

Elfin UI is part of the Elfin tool set that allows the user to design proteins with complex 3D shapes protein designs. In Elfin, a three-dimensional structure, defined as a network of nodes and edges, is translated into a protein structure using a combination of compatible structural building blocks, referred to as modules. Different module databases can be used and users can build their own, as described in the Supplementary Materials.

As an add-on, Elfin UI borrows Blender’s graphical interface to enable the generation of 3D structures to facilitate two main design processes: (1) path guide building, and (2) manual module placement.

Path guides are 3D objects, formed by nodes and edges, that describe the geometry of a three-dimensional shape. Path guides can be exported to Elfin Solver (the core algorithm in Elfin), which generates a protein structure to fit, as close as possible, the defined 3D shape.

Alternatively, protein modules, which correspond to super-secondary structural elements (e.g., sets of alpha helices and beta sheets), can be manually placed. The protein chain can be then extended by adding compatible modules, allowing for a stepwise and interactive building of protein structures.

Elfin UI introduces a new panel of options in Blender and new import and export features that enable path guide building, manual module placement and hybrid designs.

### Blender Interface

**FIGURE 2:**
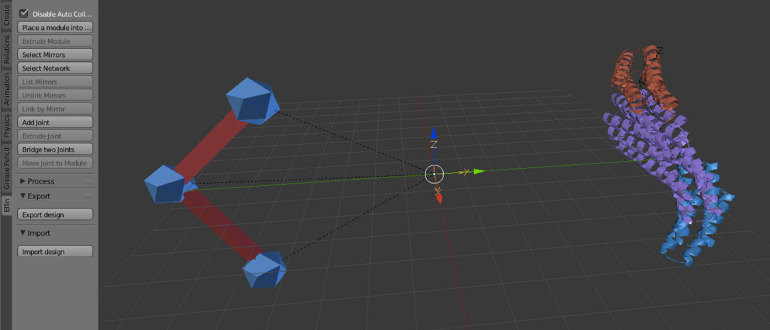
The Elfin UI Blender add-on interface. The Elfin panel on the left shows the accessible operators. On the Blender scene, on the left is a path guide composed of three joints (blue icospheres) and two bridges (red), and on the right a protein formed by three modules, in different colors.

Elfin UI specific controls are located in an “elfin” panel in the Blender interface (Figure 2). The commands, called operators, allow paths guide building and module placement. Depending on current selected objects, only allowed operators can be used. Operators are also available in the search menu, accessible using the spacebar, in Blender 2.79. Every operator has a hashtag-three-letters-shortcut that, when entered in the search menu, immediately brings up that operator, speeding up the design process. E.g., the module extrusion operator is “#exm.” Operators’ detailed descriptions are available in the Elfin UI tutorial: https://github.com/Parmeggiani-Lab/elfin-ui/blob/master/resources/tutorial/README.md. Blender operators, like delete, work on these objects.

Modules are represented by meshes, derived from PyMol (DeLano, 2002) that depict protein secondary structures (helices, beta sheets and loops) and have been scaled appropriately: each square in the reference plane of the default Blender working space is 1 nm long. Interactions and relative positions are precomputed and stored in a database file, therefore, to preserve the relationships, module scaling is not allowed.

Elfin UI allows export of path guides and designed proteins as JSON files, which contain information about connectivity, type of modules (if present) and three-dimensional coordinates. Elfin solutions, produced as JSON files, contain a network of modules and can be imported in Elfin UI for visualization. JSON was chosen for its human-readability (which facilitates debugging and easy extension), ease to parse, and because there is not a large amount of data to justify size-efficient formats, such as binary formats.

Elfin UI is a module-centric interface and does not support atom or residue level views. Atomic models, in mmCIF format, are generated from json files by a script (stitch.py) in the Elfin tool set (see Supplementary Figure S1 for details). Output files can be then visualized using molecular viewers (e.g., PyMol, Chimera, VMD) or loaded in any program that supports mmCIF files for energy minimization, molecular dynamics simulations or further design. After conversion from the modular coarse-grained representation to atomic coordinates, we perform energy minimization and relaxation in Rosetta (Leman et al., 2020) to ensure that the design shape is maintained (see Supplementary Materials).

### Path Guide Building

Path guides are the objects that guide Elfin Solver to build a protein that most resembles the user’s design intent. Path guides are not protein modules; they are simple geometry specifications expressed as “joints” and “bridges.” These are synonymous to “nodes” and “edges” in mathematical terms, and in Blender, they are represented with premade icosphere and elongated cubes respectively (Figure 2).

The main path guide operators are:

• Add joint: place a new joint in space
• Extrude joint: create a new joint in the desired position connected to the current joint with a bridge
• Bridge two joints: create a new bridge between joints

When connecting between joints, bridges will stretch and contract visually according to the actual distance between the joints. Joints and bridges can be used to define complex networks. Since the distance between joints can be arbitrarily defined, there may not always be a solution in which protein modules can satisfy the path guide design, but Elfin Solver always tries to optimize.

**FIGURE 3:**
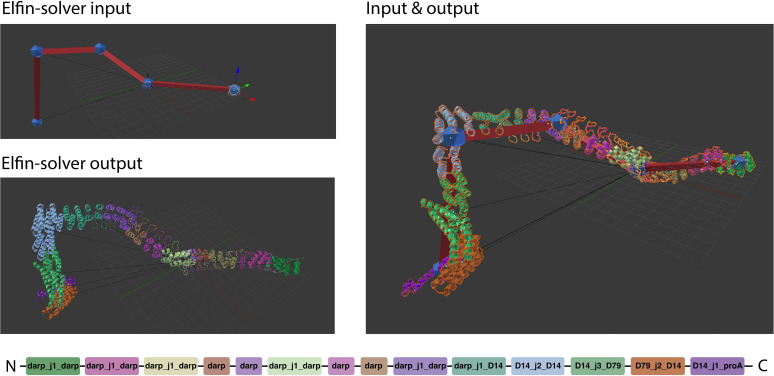
Path guide building. Elfin UI allows to define a network of joints and bridges that can be used as input for Elfin solver. The designed output can be superimposed on the initial path guide. The colors indicate the different building blocks highlighted in the sequence at the bottom.

After a design has been drawn out by the user, it can be exported into a JSON format that Elfin Solver reads and processes. The optimized solution is saved into a JSON file that Elfin UI can read back into Blender and display as a 3D model (Figure 3).

Path guides are used to define arbitrary shapes that the user is interested in. If the goal is a precise geometry in 2D or 3D, the coordinates for each node can be inputted directly in Blender.

### Manual Module Placement

**FIGURE 4:**
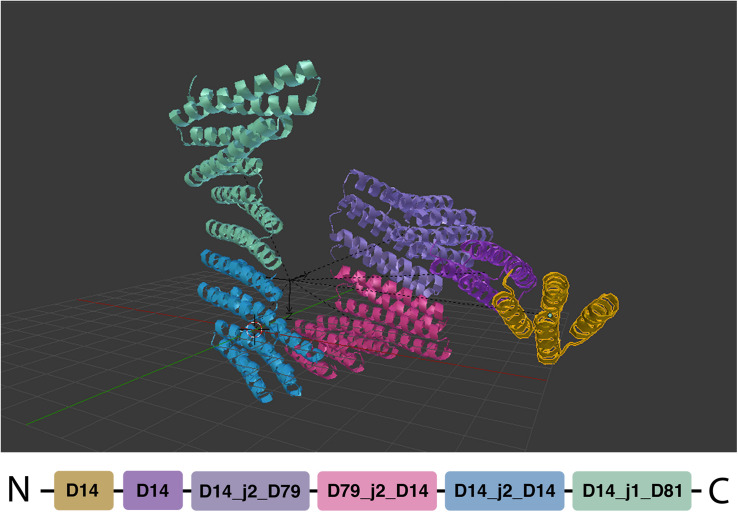
Manual module placement. The single chain protein is built as a sequence of compatible modules, depicted in different colors.

Elfin UI can be used as a sandbox environment to interactively explore the construction of complex protein architectures. Users can select modules and place them directly into the scene, growing chains progressively by addition of new compatible modules (Figure 4). When a new module is placed the color can be changed. If a new module causes clashes with the existing chains, an error box is raised, preventing the addition. This check can be disabled by toggling the auto_collision_check box in the elfin panel.

The main module operators are:

• Place modules: place a new module in the scene
• Extrude module: place a new module next to the current one extending the protein chain; the new module is selected among the compatible ones
• Link by mirror: associate two or more identical modules; when one of these modules is extruded, all the linked ones are extruded accordingly, if the extrusion is possible. Added modules are considered linked to each other
• Unlink mirror: remove the mirror linkage, so that extrusion can be performed independently.

Modules are derived from existing experimental structures (Kramer et al., 2010; Brunette et al., 2015, 2020; Wu et al., 2017; Youn et al., 2017) and connected through peptide bonds. The interfaces between modules and their relative orientation are also derived from crystal structures and SAXS-confirmed models, ensuring a correct module placement. This information is stored in the elfin and Blender databases (see section “Methods”).

**FIGURE 5:**
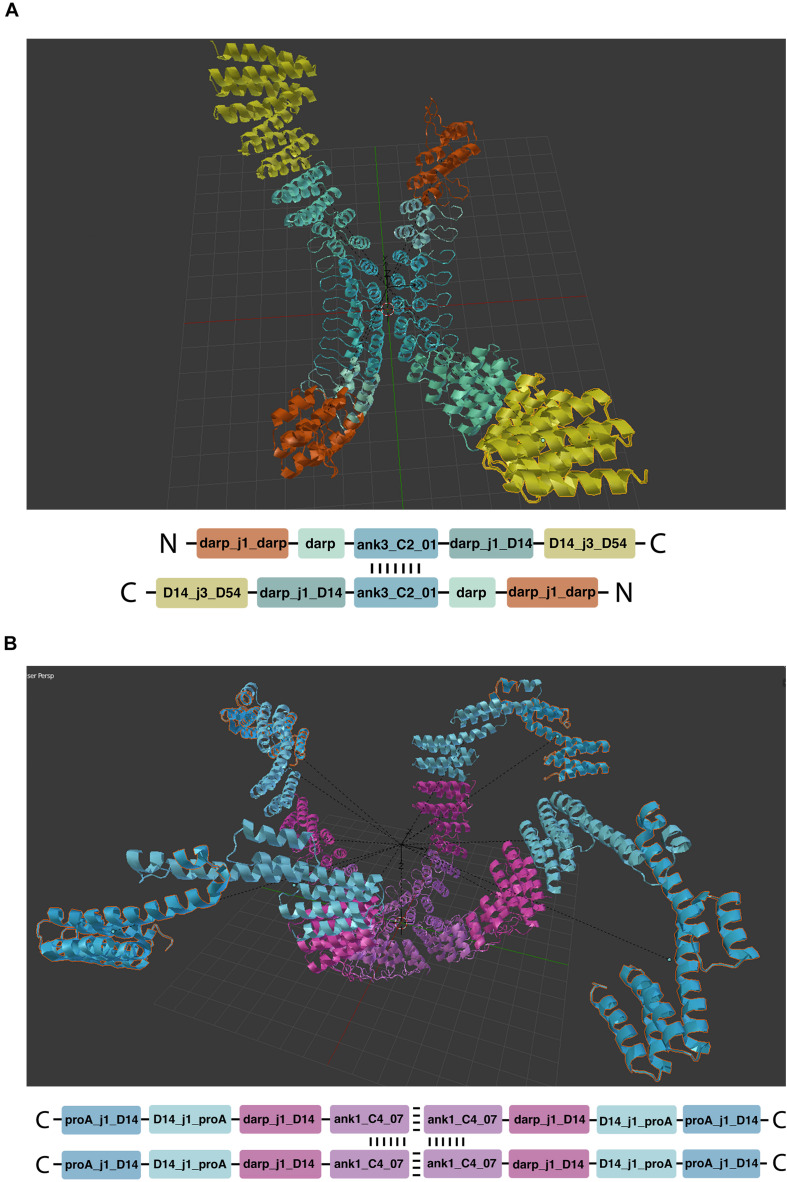
Symmetric structures. **(A,B)** Show respectively two and four chain architectures. The oligomeric module (hub) is indicated by the repeated vertical and horizontal dashes.

Mirror linking is used to build symmetric structures or structures containing only some symmetric parts (Figure 5). Mirror-linked modules need to be of the same type. Modules derived from experimentally validated oligomers (Fallas et al., 2017) contain multiple chains that can potentially grow in a symmetric fashion, when the same module is added to each chain. Symmetric hubs are automatically mirror-linked. Modules extruded from mirror-linked modules are automatically mirror-linked.

### Hybrid Design

**FIGURE 6:**
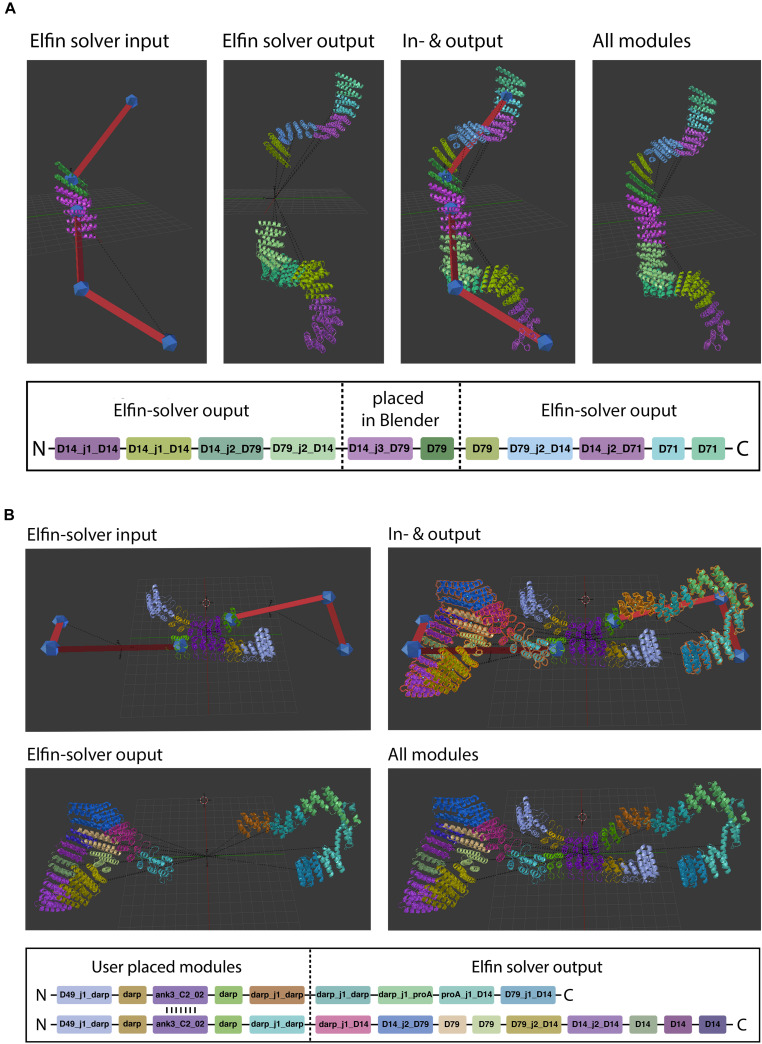
Hybrid design. Elfin UI allows users to build shapes that include selected modules in specific positions. The path guide parts are solved by Elfin solver and merged in Elfin UI. **(A,B)** Show single-chain and two-chains hybrid designs, respectively.

Manual module placement can be used in conjunction with path guides to partially define a design, if the user already knows what protein module needs to be positioned (e.g., predefined binding sites) and in which orientation (Figure 6). The user places modules directly into the scene and translates and rotates them.

When a protein module is placed directly on a path guide joint, Elfin UI infers that the bridges connecting to that joint are intended to be “extrusions” from the protein module. The “move joint to module” operator allows to place an existing joint on a module, after selecting both.

Hybrid design can be used when the position and orientation of specific modules of the desired protein are known. By building a guide path from them, elfin will search for a compatible solution to connect the modules. The initial input and the design output should be then combined in a single network, using the “join network” operator to obtain the combined structure. This approach can be used, for example, to build multivalent ligands to engage multiple cell receptors at the same time, by placing binding interfaces in the desired positions and orientation and searching for structures that can accommodate them.

### Designing With Elfin UI: Multivalent Erythropoietin Receptor Ligands

Elfin UI can be used to rapidly design rigid protein scaffolds to control the display of ligands for cell surface receptors. Dimeric ankyrin-based ligands for the erythropoietin receptor (EpoR) have been shown to induce receptor dimerization and modulate the signaling output as a function of the distance and orientation of binding sites (Mohan et al., 2019). We have used this system as a test case to assess the ability of Elfin UI to rapidly design models for alternative geometries and increased valency through manual module placement.

**FIGURE 7:**
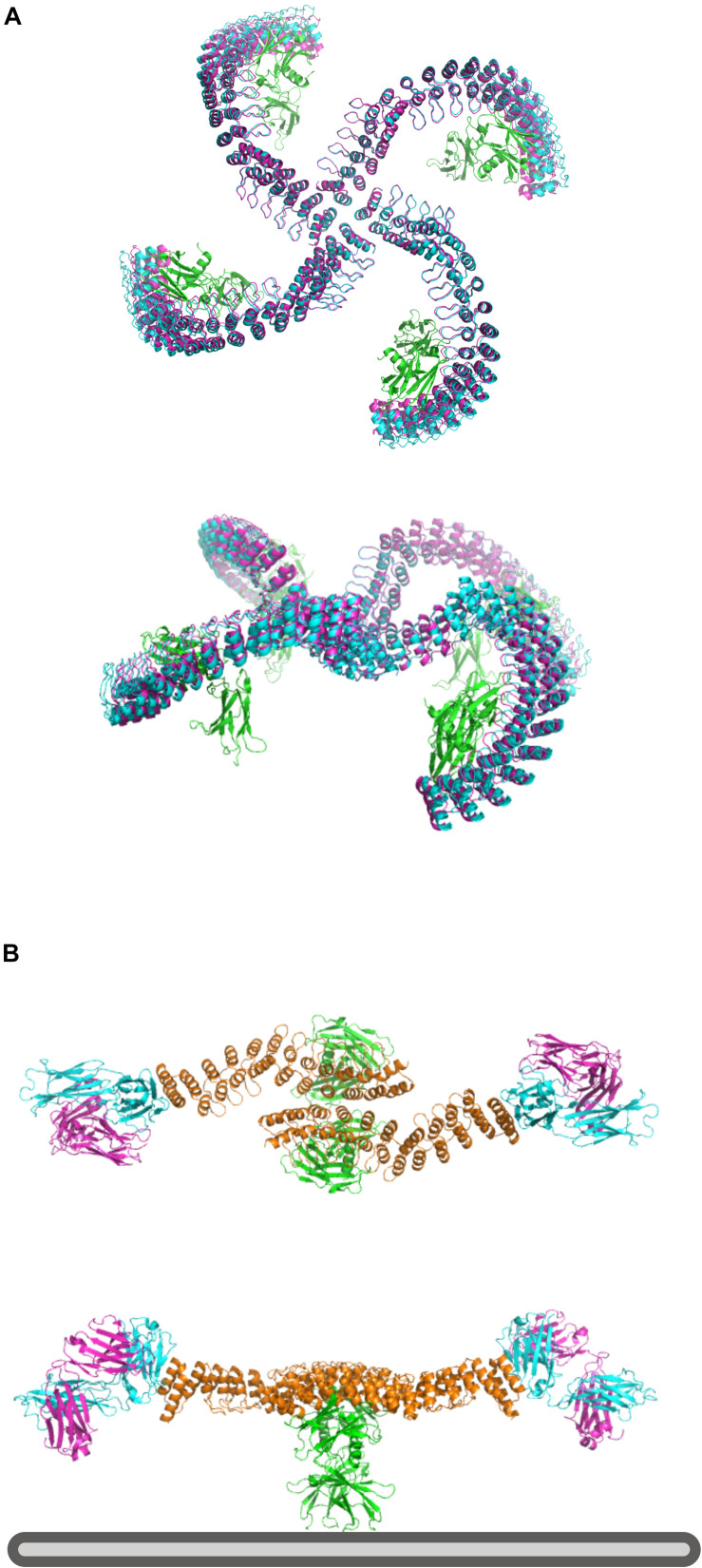
Design of multivalent ligands. **(A)** Tetramer binder for epoR, top and side view. The receptor is in green, the elfin UI design in cyan and the repacked and energy minimized model in magenta, showing only small deviation from the coarse-grained design. **(B)** Bispecific binder, top and side view. In orange is the dimeric design, in green epoR and in cyan and magenta the Fab fragment. Each design chain binds one copy of the receptor and one Fab fragment, orienting the antibody binding site toward the plasma membrane (bottom, gray) where it could engage with a target receptor of interest.

The first design has been generated by choosing a central tetrameric hub, extending it progressively, and ending with an ankyrin module that hosts the EpoR ligand, while avoiding clashes with the receptors (Figure 7A). The second model has been designed to provide multiple specificities. The scaffold contains two EpoR binding sites and two protein A domains able to bind a conserved region of a Fab antibody fragment, which can provide additional specificity for a desired cell surface target (Figure 7B). The designed structures are preserved after cycles of minimization and side chain repacking.

Each design with Elfin UI required about 1 h of work, including energy minimization and side chain repacking with Rosetta. In the second case, the Elfin UI design was used as a starting point for further engineering, shortening the proA module and moving the binding site to allow the placement of FAB in a position more compatible with multivalent binding. The output files are provided in the Supplementary Materials.

## Discussion

Elfin UI is a dedicated tool for coarse-grained design of custom protein architectures through building blocks combinations. Modular units are connected to form a single or multiple chains structure, depending on the modules used. The process is much faster than other backbone building methods, but it requires a highly curated database containing already all the possible pairs of modules in the correct orientation. Because of the nature of the database, interfaces between modules are already defined and further sequence design is not needed, contributing to improve the design speed, both in terms of automated solutions and feedback to users that build structures interactively. However, repacking and energy minimization are recommended to eliminate small discrepancies at the connection points between modules. External software tools (e.g. Rosetta) are required for modifications at atomic level, including repacking, energy minimization and point mutations.

Elfin UI represents a new type of interactive design software for protein design. While other tools traditionally operate directly on atomic models, Elfin UI allows the user to act at a higher level, enabling a rapid design for a desired shape which is not arbitrary, but it is connected to the information in the module database. Quality, size and fit to the design task of the database are key factors for successful designs. The precomputed database is one of the factors influencing design speed, together with the visualization of our modules, which are represented by rigid meshes, appearing in blender as full-fledged secondary structures. Moreover, all calculations (e.g., collision detection, partial overlap, distance) are performed with each module considered as a sphere with defined radius, therefore drastically reducing the computational costs.

This setup allows for rapid prototyping of potential structures of interest, exploring sequences with different lengths and shape. The option to generate custom databases allows for greater flexibility in cases where only specific types of modules could be used, e.g., peptide or protein binding domains.

Elfin UI’s intuitive approach makes protein design of novel protein structures, and in particular large custom scaffolds, accessible to non-experts and to the general public, and represents a new educational and outreach tool.

Precise and reliable design of biological systems is one of the goals of synthetic biology. With Elfin, custom structures with functional domains in specific positions and orientations can be easily and rapidly designed, bringing proteins into the realm of DNA nanotechnology.

## Data Availability Statement

Publicly available datasets were analyzed in this study. This data can be found here: https://github.com/Parmeggiani-Lab/elfin.

## Author Contributions

C-TY wrote the software. LO tested and optimized the software. FP devised and supervised the project and wrote the manuscript. All the authors read and commented on the manuscript.

## Conflict of Interest

The authors declare that the research was conducted in the absence of any commercial or financial relationships that could be construed as a potential conflict of interest.
