# Supplementary material for: Elfin UI: A Graphical Interface for Protein Design With Modular Building Blocks
Source: Front Bioeng Biotechnol. 2020 Oct 23;8:568318. doi: 10.3389/fbioe.2020.568318 (PMC7644802; doi:10.3389/fbioe.2020.568318)
Supplement: Supplementary file 1 [file Data_Sheet_1.pdf]

Elfin UI: a graphical interface for protein design with modular building blocks

Authors: Chun-Ting Yeh[1], Leon Obendorf[1,2], Fabio Parmeggiani[1,3]

1 School of Chemistry and School of Biochemistry, University of Bristol, Bristol, UK

2 Institute of Chemistry and Biochemistry, Freie Universität Berlin, Berlin, Germany

3 Bristol Biodesign Institute and BrisSynBio, a BBSRC/EPSRC Synthetic Biology Research Centre, University of Bristol, Bristol, UK

Supplementary materials

1) Converting designs from coarse grained (JSON) to atomic coordinates (mmCIF)

Elfin and Elfin UI are design platforms that operate at coarse grained level, with modules as fundamental unit. Once a design is completed, the resulting JSON file needs to be converted in an atomistic model for further use.

Fig.1s shows a simple three-dimensional coordinate transformation stage. Once a design is complete (either fully specified via Elfin-UI, or with path guides solved by Elfin-Solver), it can be exported by Elfin-UI in JSON format (chosen for readability). The JSON file describes what proteins are to be placed where (translation), facing which way (rotation), and connects with which neighbours (linkage information), as shown by the leftmost portion of Fig.1s. The schema also supports multiple chains.

Elfin's Python output script (stitch.py, located in the /elfin/elfinpy/ directory) then references the protein database for atom coordinates for each module required in the design. It then projects the required atoms according to the translation (T) and rotation (R) matrices stored in the JSON file. Lastly, free termini are "capped" by adding capping module residues which are sequences responsible to shield the otherwise exposed hydrophobic core of modules and increase the solubility of proteins. Like all modules, also capping units are derived from experimentally verified structures. Ensuring each atom belongs to the correct residue and chain, the overall atom structure is then written to a mmCIF file.

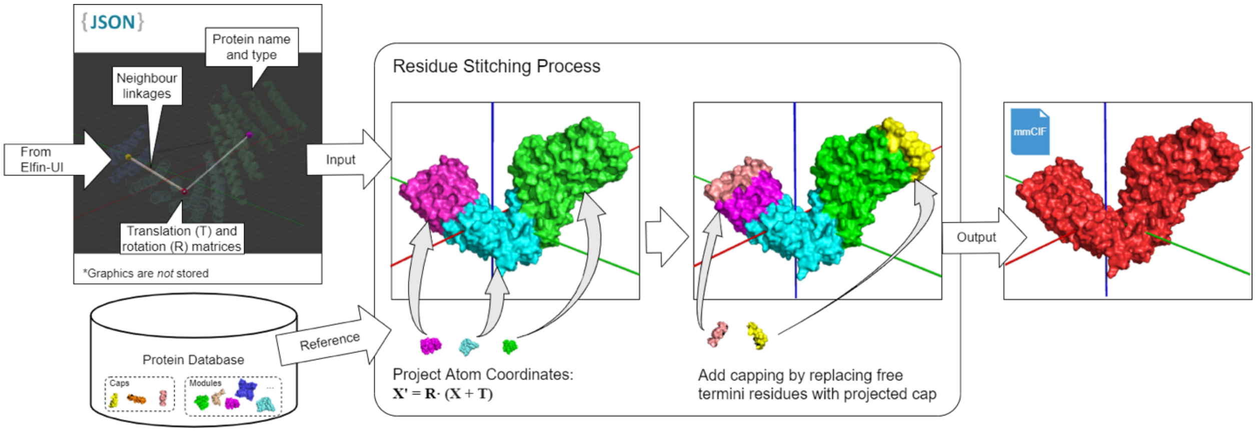

Fig.1s. An illustration of how the design data exported by Elfin-UI gets transformed into an atomic model in mmCIF format.

2) Structure validation using Rosetta

Atomic models, derived from the conversion of coarse-grained modular designs, need to be energy minimized and validated with an appropriate forcefield. We use commonly the Rosetta modeling suite, and in particular the rosetta_script application, for its speed and ability to handle symmetry, if present.

rosetta_script -s <full atom mmCIF file> -parser:protocol minCart_relax.xml

The executable name will differ slightly depending on the operating system, mac or linux

The xml script minCart_relax.xml, below, provides instructions for energy minimization and relaxation.

```
<ROSETTASCRIPTS>
    <SCOREFXNS>
          <ScoreFunction name="cart" weights="ref2015_cart"/>
    </SCOREFXNS>
    <RESIDUE_SELECTORS>
    </RESIDUE_SELECTORS>
    <TASKOPERATIONS>
          <IncludeCurrent name="IC" />
    </TASKOPERATIONS>
    <FILTERS>
    </FILTERS>
    <MOVERS>
      <FastRelax name="relax" task_operations="IC"/>
      <MinMover name="min_cart" scorefxn="cart" chi="0" bb="1"
cartesian="true"/>
    </MOVERS>
    <PROTOCOLS>
      <Add mover_name="min_cart"/>
      <Add mover_name="relax"/>
    </PROTOCOLS>
    <OUTPUT />
</ROSETTASCRIPTS>
```

Energy minimization in cartesian space (xyz coordinates) allows to efficiently minimize the peptide bonds between modules and avoid chain breaks.

Relax combines a series of side chain repacking and energy minimization that operate on structures torsion angles rather than cartesian coordinates.

For symmetric systems with cyclic symmetry and symmetry axis aligned to z axis, the xml script minCart_relax_sym.xml below can be used.

```
<ROSETTASCRIPTS>
    <SCOREFXNS>
          <ScoreFunction name="cart" weights="ref2015_cart"/>
    </SCOREFXNS>
    <RESIDUE_SELECTORS>
    </RESIDUE_SELECTORS>
    <TASKOPERATIONS>
          <IncludeCurrent name="IC" />
    </TASKOPERATIONS>
    <FILTERS>
    </FILTERS>
    <MOVERS>
      <DetectSymmetry name="detect"/>
      <SymMinMover name="min_sym_cart" scorefxn="cart" bb="1" chi="0"
jump="ALL" cartesian="true"/>
      <FastRelax name="relax" task_operations="IC"/>
    </MOVERS>
    <PROTOCOLS>
      <Add mover_name="detect"/>
      <Add mover_name="min_sym_cart"/>
      <Add mover_name="relax"/>
    </PROTOCOLS>
    <OUTPUT />
</ROSETTASCRIPTS>
```

3) Modules and sources

Currently available modules are listed in Table 1s. The references indicate the source publications. The modules included in the library are derived from experimentally validated structures, either by x-ray crystallography (with PDB ID indicated) or small angle x-ray scattering (SAXS). Capping units, not indicated in the table, are based on core modules and derived from the same original structures. Designed Helical Repeats (DHR) are de novo repeats. ProA is a domain from Stapphilococcus aureus protein A that has been engineered in a repeat protein format. DARPins (darp), ank1, ank3 and ank4 are similar consensus designs for the ankyrin repeat protein family. They contain minor differences, but the overall structure is conserved. The darp interface between repeats has been used as reference and the other ankyrin modules have been mutated to provide fully compatible interfaces.

Table 1s

Module type	Name	Reference	Method	Structure type
**Core**	D4	Brunette et al., 2015	5CWB, SAXS	DHR
	D8	Brunette et al., 2015	5CWF, SAXS	DHR
	D14	Brunette et al., 2015	5CWH, SAXS	DHR
	D18	Brunette et al., 2015	5CWI, SAXS	DHR
	D49	Brunette et al., 2015	5CWJ, SAXS	DHR
	D53	Brunette et al., 2015	5CWK, SAXS	DHR
	D54	Brunette et al., 2015	5CWL, SAXS	DHR
	D64	Brunette et al., 2015	5CWM, SAXS	DHR
	D71	Brunette et al., 2015	5CWN, SAXS	DHR
	D76	Brunette et al., 2015	5CWO, SAXS	DHR
	D79	Brunette et al., 2015	5CWP, SAXS	DHR
	D81	Brunette et al., 2015	5CWQ, SAXS	DHR
	darp	Kramer et. al., 2010	2XEE	Consensus ankyrin
	proA	Youn et. al., 2017	5H7A	Protein A domain
**Junctions**	D4_j1_D64	Brunette et al., 2020	SAXS	DHR - DHR
	D14_j1_D14	Brunette et al., 2020	SAXS	DHR - DHR
	D14_j1_D18	Brunette et al., 2020	6W2V, SAXS	DHR - DHR
	D14_j1_D54	Brunette et al., 2020	SAXS	DHR - DHR
	D14_j1_D76	Brunette et al., 2020	SAXS	DHR - DHR
	D14_j1_D79	Brunette et al., 2020	SAXS	DHR - DHR
	D14_j1_D81	Brunette et al., 2020	SAXS	DHR - DHR
	D14_j1_proA	Youn et. al., 2017	5H7C	DHR - proA
	D14_j2_D8	Brunette et al., 2020	SAXS	DHR - DHR
	D14_j2_D14	Brunette et al., 2020	SAXS	DHR - DHR
	D14_j2_D18	Brunette et al., 2020	6W2W, SAXS	DHR - DHR
	D14_j2_D54	Brunette et al., 2020	SAXS	DHR - DHR
	D14_j2_D71	Brunette et al., 2020	SAXS	DHR - DHR
	D14_j2_D79	Brunette et al., 2020	SAXS	DHR - DHR
	D14_j3_D8	Brunette et al., 2020	SAXS	DHR - DHR
	D14_j3_D54	Brunette et al., 2020	SAXS	DHR - DHR

	D14_j3_D79	Brunette et al., 2020	SAXS	DHR - DHR
	D14_j4_D79	Brunette et al., 2020	SAXS	DHR - DHR
	D14_j5_D79	Brunette et al., 2020	SAXS	DHR - DHR
	D18_j1_D14	Brunette et al., 2020	SAXS	DHR - DHR
	D49_j1_D14	Brunette et al., 2020	SAXS	DHR - DHR
	D49_j1_D79	Brunette et al., 2020	SAXS	DHR - DHR
	D49_j1_D81	Brunette et al., 2020	SAXS	DHR - DHR
	D49_j1_darp	Ozture, Harrison, Parmeggiani, unpublished	SAXS	DHR - ankyrin
	D53_j1_D4	Brunette et al., 2020	6W2Q, SAXS	DHR - DHR
	D53_j1_D79	Brunette et al., 2020	SAXS	DHR - DHR
	D54_j1_D79	Brunette et al., 2020	6W2R, SAXS	DHR - DHR
	D79_j1_D54	Brunette et al., 2020	SAXS	DHR - DHR
	D79_j2_D14	Brunette et al., 2020	SAXS	DHR - DHR
	darp_j1_D14	Ozture, Harrison, Parmeggiani, unpublished	SAXS	Ankyrin - DHR
	darp_j1_darp	Wu et. al., 2017	5LEB	Ankyrin - ankyrin
	darp_j1_proA	Youn et. al., 2017	5H76	Ankyrin - proA
	proA_j1_D14	Youn et. al., 2017	5H7C	proA - DHR
**Hubs**	ank3_C2_02	Fallas et al., 2017	SAXS	Ankyrin homodimer
	D4_C4_G1	Fallas et al., 2017	SAXS	DHR homotetramer
	D79_C2_VO1	Fallas et al., 2017	SAXS	DHR homodimer
	ank4_C4_02	Fallas et al., 2017	5KWD, SAXS	Ankyrin homotetramer
	ank3_C2_01	Fallas et al., 2017	5HRY, SAXS	Ankyrin homodimer
	ank1_C4_07	Fallas et al., 2017	5HS0, SAXS	Ankyrin homotetramer
	ank1_C2_G3	Fallas et al., 2017	5KBA, SAXS	Ankyrin homodimer

4) Custom database creation

Elfin library can be expanded by adding modules, or by generating entirely new libraries. Fig.2s shows the process of preparing structures to add to the library. A detailed step by step guide with commands is described in the elfin documentation on github.

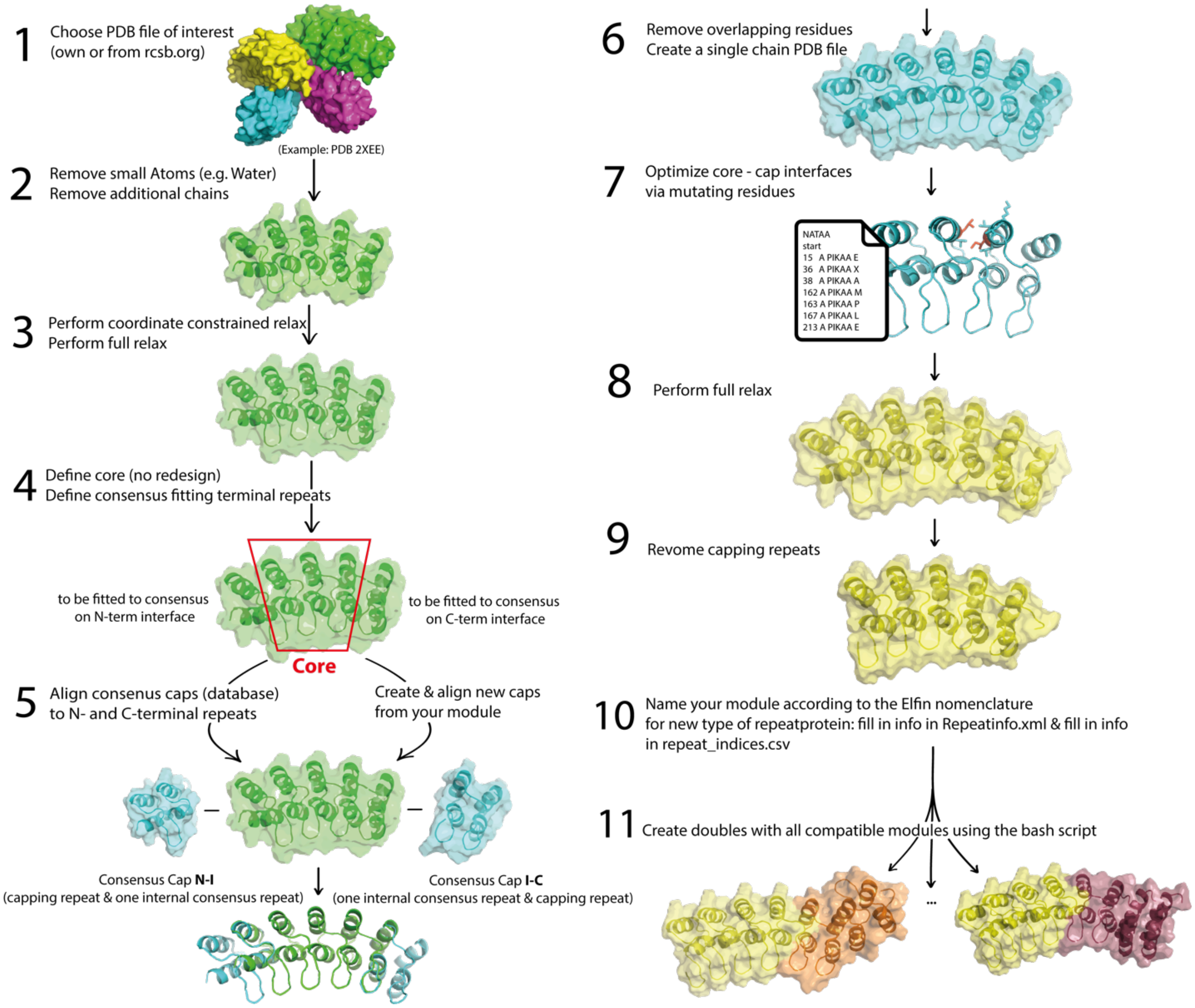

Figure 2s. Preparation of new modules for elfin libraries. The diagram illustrates the steps for preparing an ankyrin module. 1-3) Selection and preparation of structure: we use Rosetta to remove unwanted molecules and perform energy minimization and repacking. 4-5) Define overlapping regions: we determine what is the part of the structure that will be the module (e.g. a core one) and what are the capping repeats. Overlapping regions for future alignment are then defined: e.g. in the case of capping repeat, an extra internal repeat (I) is added to allow for superposition with the module. 6-9) When the new module needs to be connected with pre-existing modules, mutations and relaxation are performed with Rosetta to ensure compatibility. 10-11) Name and compatibility information are assigned, and all the potential pairs within the database are generated.

5) Supplementary data

Json and blender files for the examples in the article are provided in the compressed supplementary_data.zip.

design_Fig3

design_Fig4

design_Fig5a

design_Fig5b

design_Fig6a

design_Fig6b

design_Fig7a

design_Fig7b

For the two Fig7 designs, consensus ankyrin modules were used as placeholder for binding interfaces. mmcif and pdb files after relaxation (mrs_ files) are included in the supplementary data.

The structure of design_Fig7b was modified after the initial elfin UI design, to adjust the position of the Fab binding interface. The darp_j1_proA module, contains two proA motives, of which the first overlapping with the fusion helix between darpin and proA units. The second motif was removed and the sequence of the first mutated to account for the position at the C-terminal of the protein.
